# Sensory evaluation and consumer acceptance of naturally and lactic acid bacteria-fermented pastes of soybeans and soybean–maize blends

**DOI:** 10.1002/fsn3.82

**Published:** 2013-12-20

**Authors:** Tinna A Ng'ong'ola-Manani, Agnes M Mwangwela, Reidar B Schüller, Hilde M Østlie, Trude Wicklund

**Affiliations:** 1 Department of Food Science and Technology, Lilongwe University of Agriculture and Natural ResourcesBunda College Campus, PO Box 219, Lilongwe, Malawi; 2 Department of Chemistry, Biotechnology and Food Science, Norwegian University of Life SciencesPO Box 5003, Ås, 1430, Norway

**Keywords:** Drivers of liking, lactic acid bacteria fermentation, natural fermentation, preference mapping, soybean pastes

## Abstract

Fermented pastes of soybeans and soybean–maize blends were evaluated to determine sensory properties driving consumer liking. Pastes composed of 100% soybeans, 90% soybeans and 10% maize, and 75% soybeans and 25% maize were naturally fermented (NFP), and lactic acid bacteria fermented (LFP). Lactic acid bacteria fermentation was achieved through backslopping using a fermented cereal gruel, *thobwa*. Ten trained panelists evaluated intensities of 34 descriptors, of which 27 were significantly different (*P* < 0.05). The LFP were strong in brown color, sourness, umami, roasted soybean-and maize-associated aromas, and sogginess while NFP had high intensities of yellow color, pH, raw soybean, and rancid odors, fried egg, and fermented aromas and softness. Although there was consumer (*n* = 150) heterogeneity in preference, external preference mapping showed that most consumers preferred NFP. Drivers of liking of NFP samples were softness, pH, fermented aroma, sweetness, fried egg aroma, fried egg-like appearance, raw soybean, and rancid odors. Optimization of the desirable properties of the pastes would increase utilization and acceptance of fermented soybeans.

## Introduction

Diets of most rural Malawian households are poorly diversified and are predominantly maize-based. Maize contributes to over 60% of energy, total iron, zinc, riboflavin, and about half of protein consumption, when animal-source foods are scarce (Ecker and Qaim 2011). Yet, maize has low protein content (9.42%) and is limited in micronutrients (Nuss and Tanumihardjo 2010). Such maize-based diets increase the risk of various types of malnutrition. In Malawi, the prevalence of chronic malnutrition among under-5 children is high, that is 47% (National Statistics Office and ICF Macro 2011), and micronutrient deficiencies were reported among under-5 children, women, and men (National Statistics Office and Macro 2005). Malnutrition in Malawi is attributed to insufficient energy and nutrient intake, among other factors (Maleta 2006). Animal-source foods provide good quantities of protein and other nutrients, but they are expensive. This calls for alternative low-cost source of nutrient-dense food that can be consumed by adults and children.

Legumes, including soybeans (*Glycine max*), provide good quantities of protein, riboflavin, calcium, and iron (Messina 1999). Soybeans have been used in the prevention and treatment of protein energy malnutrition in young children, and in improving the nutritional status of communities. Therefore, soybean is a good alternative to expensive animal-source proteins (United Nations Industrial Development Organization 2003). In Malawi, soybean is produced mainly as a cash crop with limited household-based consumption (CYE Consult 2009; Tinsley 2009). Production increased over the past 5 years and in 2010, 73,000 tonnes of soybeans were produced. Most of the soybeans (63,000 tonnes) were used within the country. However, the demand for production is driven by the poultry feed industry (Markets and Economic Research Centre of the National Agricultural Marketing Council 2011) while there is limited demand from the corn–soy blend industry (Tinsley 2009). Unfortunately, there is no statistics indicating the percent consumption of both industries. Nevertheless, various reports show that direct human consumption of soybeans in Malawian households is through enriched maize flour containing up to 20% soybean flour (Katona-Apte 1993; Kalimbira et al. 2004; Maleta 2006; CYE Consult 2009; Tinsley 2009). The enriched flour locally known as *Likuni Phala* is used as a weaning food in children (Kalimbira et al. 2004; Maleta 2006; CYE Consult 2009) and is also distributed by nongovernmental organizations for school feeding programs, for hospitals, orphanages, and refugee camp usage (Katona-Apte 1993; Tinsley 2009). Consumption of maize together with soybeans provide high-quality protein diet comparable to diets containing animal protein (Asgar et al. 2010), because limiting amino acids in maize are complemented by those found in soybeans (Siegel and Fawcett 1976; FAO 1992).

Despite the nutritional benefits, household soybean utilization in Malawi is still minimal due to limited knowledge in processing (Coulibaly et al. 2009). Processing is required to eliminate antinutritional factors and the undesirable characteristic “beany” taste. Various processing methods such as boiling, steaming, roasting, germination, fermentation, and milling improve soybean utilization (Siegel and Fawcett 1976; Anderson and Wolf 1995; Golbitz 1995; Wang and Murphy 1996). Use of fermented soybean products in Asia is widely documented (Sarkar et al. 1994; Kwon et al. 2010; Dajanta et al. 2012; Park et al. 2012).

In order to increase direct household consumption of soybeans in Malawian diets, pastes of fermented soybeans and soybean–maize blends were developed as an alternative low-cost source of protein. The pastes were naturally fermented or lactic acid bacteria (LAB) fermented through backslopping using a traditional fermented cereal gruel, *thobwa*. The developed pastes were to be used as side dishes, such as in *kinema* (Sarkar et al. 1994) and other similar products of the Orient. Most soybean-fermented products are naturally fermented by *Bacillus subtilis* (Steinkraus 1997), a proteolytic microorganism that produces ammonia during fermentation (Sarkar and Tamang 1995; Dakwa et al. 2005). High amounts of ammonia result in strong odor, which some people find objectionable (Allagheny et al. 1996; Parkouda et al. 2009). LAB fermentations, on the other hand, improve flavor of traditional foods (Steinkraus 1997).

The developed products were new to Malawian consumers; therefore, it was important to obtain consumer feedback for improvement of the products. Preference mapping (PREFMAP) techniques were used to find out the potential of the developed products for future use and to determine the sensory properties driving consumer preferences. PREFMAP techniques have been widely used in different food products (Helgesen et al. 1997; Lawlor and Delahunty 2000; Guinard et al. 2001; Thompson et al. 2004) to understand sensory attributes that drive consumer acceptability (Murray and Delahunty 2000; Thompson et al. 2004; van Kleef et al. 2006; Dooley et al. 2010; Resano et al. 2010). Thus, the objectives of this study were to describe sensory properties of the fermented pastes, to determine consumer acceptance of the pastes, and to find out sensory properties that drive acceptance of the pastes.

## Material and Methods

### Preparation of pastes of soybeans and soybean–maize blends

Pastes of soybeans and soybean–maize blends were prepared in the laboratory. Soybeans (Nasoko, variety code 427/6/7) were sorted, washed, and boiled for 30 min and dehulled by rubbing between palms in cold water, washed again, and then boiled for 1 h (Dakwa et al. 2005). Maize (DK8071) was boiled for 2 h (to make it soft) before being ground together with soybeans into a paste. Grinding was done for 10–15 min in a Waring Commercial blender (800ES; Waring, Torrington, CT), which was sterilized by boiling for 5 min. Sterile water (100 mL) was added during the grinding to make the pastes. LAB fermentation was facilitated by the addition of fermented maize and finger millet (*Eleusine coracana*) gruel (*thobwa*). The preparation of *thobwa* was according to Kitabatake et al. (2003). Pastes for LAB fermentation (LFP) were backslopped (BS) using 10% (v/w) *thobwa*. The pH of the *thobwa* was around 4.5 with a LAB population of 10^8^ cfu/mL. Naturally fermented pastes (NFP) were made by similar treatments but without adding the fermented gruel. Paste composition was determined based on preliminary laboratory trials whereby pastes containing 100%, 75%, and 50% soybeans (the remaining proportions being maize) were studied. The preliminary study showed no significant differences in pH reduction and microbial loads (total aerobic count and LAB count) in pastes containing 75% and 50% soybeans. Thus for the study, pastes were prepared according to the following compositions: pastes of soybeans only; pastes of soybean and maize blends containing 90% and 75% soybeans. NFP were designated as 100S, 90S, and 75S according to 100%, 90%, and 75% soybean composition in the pastes, the remaining proportions being maize. Similarly, BS LAB-fermented pastes were designated 100SBS, 90SBS, and 75SBS. Portions of 500 g for all treatments were fermented at 30°C for 72 h in glass jars.

### Analyses of chemical and physical properties

Titratable acidity (g lactic acid/100 g sample) and pH were determined according to AOAC (1990). The pH was measured using a pH meter (WTW pH 525; D. Jurgens and Co., Bremen, Germany) fitted with a glass electrode (WTW SenTix 97T). Amino acids were extracted from freeze-dried homogenized samples and were determined using High-performance liquid chromatography according to Bütikofer and Ardö (1999). Salt content was determined using a Sherwood MK II Chloride Analyzer (Model 926; Sherwood Scientific Ltd., Cambridge, U.K.) according to the manufacturer's operating instructions. Freeze-dried samples (1.00 g) were mixed with 20 mL of distilled water. The mixtures were heated to 55°C for 30 min and were filtered before chloride analysis. Viscoelastic properties of the samples were analyzed using a Physica MCR301 rheometer (Paar Physica, Antony Paar, Germany) fitted with a 50-mm plate/plate geometry, PP50. The temperature was kept at 20°C by the Peltier control of the bottom plate. The sample was placed on the bottom plate and gently compressed. The gap was ∼3 mm, and a constant normal force of 5 N was applied to the sample while testing took place. Amplitude sweeps were then done in oscillation at a frequency of 1 Hz varying the amplitude from 0.01% to 110% strain.

### Descriptive sensory analysis

#### Panel selection and training

Ten people interested in sensory evaluation of the fermented pastes were recruited among Nutrition and Food Science students in the Department of Home Economics and Human Nutrition; and staff members at Lilongwe University of Agriculture and Natural Resources, Bunda College campus. Panelists with ability to discriminate five tastes (salty, sweet, sour, umami, and bitter) in a solution system were selected by conducting five sets of directional paired comparison tests. Four men and six women in the age range of 20–32 years were selected as panelists. Consensus training as explained by Lawless and Heymann (1998) was conducted. Panelists were exposed to soybean-fermented pastes to be tested in the descriptive analysis sessions. Through consensus, panelists generated terms (descriptors) and definitions to describe the sensory differences among the samples. Panelists also decided on words to anchor the descriptive terms and some reference standards to be used. Trial evaluations were performed to enable decision on panelists' reproducibility. Thirty-four descriptors/attributes describing appearance, aroma/odor, taste, and texture were generated. The descriptors, their meanings, and the reference standards used are presented in Table 1. Four training sessions per week were held for 1.5 months and each session lasted ∼1 h 30 min.

**Table 1:** Descriptors and definitions used to explain sensory characteristics of the fermented pastes.

Descriptors	Abbreviations	Meanings of the descriptors	Reference/standards used
Appearance			
Brown	Brown	Intensity of brown color of the fried pastes	Color wheel
Yellow	Yellow	Intensity of the yellow color of the fried pastes	Color wheel
Fried egg-like	EggL	Appearance associated with fried egg	Fried egg
*Chitumbuwa*-like	ChituL	Appearance associated with a local snack, *chitumbuwa,* made from deep frying maize flour batter	*Chitumbuwa*
*Mandazi*-like	MandL	Appearance associated with local fritters, *mandazi*, made from deep frying wheat flour batter	*Mandazi*
Aroma/odors			
Raw soybean odor	RawS	Characteristic soybean odor strong in soymilk made from raw soybeans hydrated in cold water	Raw soymilk
Roasted soybean aroma	RoastS	Aroma associated with roasted soybean	Crushed roasted soybean
Burnt roasted soybean odor	BRoastS	Odor associated with burnt roasted soybean	Crushed burnt roasted soybean
Roasted maize aroma	RoastM	Aroma associated with roasted dried maize	Crushed roasted maize
Burnt roasted maize odor	BRoastM	Odor associated with burnt roasted dried maize	Crushed burnt roasted maize
Soaked burnt roasted maize odor	SBRoastM	Odor associated with soaked burnt roasted dried maize	Soaked burnt roasted maize
*Chitumbuwa* aroma	ChituA	Aroma associated with a local snack, *chitumbuwa,* made from deep frying maize flour batter	*Chitumbuwa*
*Mandazi* aroma	MandA	Aroma associated with local fritters, *mandazi,* made from deep frying bread flour batter	*Mandazi*
*Chigumuyoyo* aroma	Chigumu	Aroma associated with a local snack, *chigumuyoyo,* made from baking maize flour batter	*Chigumuyoyo*
Fried egg aroma	EggA	Aroma associated with fried egg	Fried egg
Fermented aroma	FermA	Aroma associated with fermented cereals	Sugar solution (20%) fermented for 24 h by 1.5 g yeast.
*Matsukwa* odor	*Matsukwa*	Odor associated with water for soaking degermed maize	Water from degermed maize soaked for 2 days
*Kondoole* aroma	*Kondoole*	Aroma associated with fermented cassava, *kondoole*	Fermented cassava
*Thobwa* aroma	*Thobwa*	Aroma associated with a local fermented beverage “*thobwa”*	*Thobwa*
*Chambiko* aroma	*Chambiko*	Aroma associated with fermented milk, *chambiko*	Commercially available *Chambiko*'
Fermented beans aroma	FBeans	Aroma associated with fermented kidney beans	Cooked beans fermented for 24 h
*Mafuta a chiwisi* odor	*Chiwisi*	Odor associated with partially heated cooking oil	Soybean cooking oil heated at 100°C for 2 min
Rancid odor	Rancid	Odor associated with rancid oil	Soybean cooking oil reused more than three times
Texture			
Softness	Soft	Amount of force necessary to compress the sample when pressed between fingers	No standard
Easiness to break	Brittle	How easy it was to break the sample (brittle)	Toasted bread, intensity = 15
Surface roughness	Rough	Irregularities on the surface or not a smooth surface	Custard pudding = 1 intensity
Graininess	Grainy	Size of the grains seen inside the sample when broken	Maize and soy grains; whole = 15 and half = 7 intensity
Sogginess	Soggy	Tendency of the sample to absorb oil as observed by pressing the sample between white paper	Comparison of amount of oil absorbed on white paper
Taste			
Sourness	Sour	Taste sensation typical of organic acids	Citric acid; 0.05% = 2 intensity, 0.2% = 15 intensity
Sweetness	Sweet	Taste sensation typical of sucrose solution	Sucrose solution; 2% = 2 intensity and 16% = 15 intensity
Saltiness	Salty	Taste sensation typical of sodium chloride	Sodium chloride; 0.2% = 2 intensity and 1.5% = 15 intensity
Bitterness	Bitter	Taste sensation typical of caffeine and quinine	0.01% quinine sulfate solution
Umami	Umami	Taste sensation typical of monosodium glutamate (MSG)	MSG solution; 0.3% = 3 intensity, 0.7% = 7 intensity
Aftertaste	AfterT	Taste lingering on tongue after sample is removed	Similar to unripe banana taste

#### Sample preparation and presentation

Maize starch (1%, w/w) was added to the fermented pastes to prevent crumbling during frying. The pastes were molded into rounds ca. 5 g each, and were fried in heated (180–195°C) soybean oil for 3–5 min. Fresh oil was used for each sample. One hour before sensory evaluation, four pieces of 5 g of each fried sample were transferred to a separate glass serving container before covering with aluminum foil. Each sample was coded with a three-digit random number and the samples were presented in random order to the panelists for evaluation. The temperature of the samples at the time of evaluation was room temperature (around 25°C).

#### Descriptive analysis procedure

Ten panelists were trained to rate attribute intensities of the six products using a 15-point unstructured line scale labeled with either “none, weak, or least” as point 1 and “very strong” as point 15. Each panelist evaluated the products individually. Products were evaluated in three sessions and all products were served at each session, hence the sessions acted as replicates. Tap water was provided to panelists to rinse their palate before and between tasting. The evaluation was conducted in a well-ventilated laboratory fitted with fluorescent lights. The temperature in the evaluation room was between 23°C and 25°C.

### Consumer acceptability test

A total of 150 consumers interested in participating in the study were recruited from three villages that participated in a nutrition, health, and agriculture project in Lungwena extension planning area, Mangochi, Malawi. Products were prepared and presented in the same way as in the descriptive analysis except that 1% (w/w) of salt was added prior to frying. Salt was added based on consumer recommendations during a questionnaire pretesting. Products were presented one at a time in a random order. The samples were coded with three-digit random numbers and served in a central location.

Consumers evaluated acceptance on taste, smell, color, smoothness, and overall acceptance of the six products using a 7-point facial hedonic scale. On the scale, point 1 referred to dislike extremely and 7 referred to like extremely, 4 was neither like nor dislike and was in the middle. Consumers were instructed on how to use the scale. Consumers were instructed to sniff and taste a sample. They were also allowed to re-taste and change their previous scores, if needed. Tap water was provided to consumers to rinse their palate before and between tasting.

### Statistical analysis

During training, panelists' reproducibility was determined using analysis of variance (ANOVA) at *P* = 0.05. Scores of each panelist were compared with the rest of the panelists for each sample. When significant differences were found, Duncan's test was performed to identify panelists that differed and the specific descriptors they scored differently. Panelists who were not reproducible were assisted to improve performance. Panel consensus was checked using profile plots generated from PanelCheck. At the end of the descriptive analysis, PanelCheck was used to assess panelists' consensus and discrimination ability of the attributes (Tomic et al. 2010). Mean intensity scores of the descriptors were compared using three-way ANOVA and least square difference test (*P* = 0.05) as post hoc, with panelists, replicates, and products as factors. Correlations between sensory attributes were also obtained.

To understand sensory attributes that characterized the products, principal component analysis (PCA) was performed. In order to identify attributes driving consumer liking, external PREFMAP was obtained by performing a partial least squares regression (PLSR) analysis. Mean intensity scores of attributes that were significantly different (*P* < 0.05) on product effect and mean values of chemical and physical properties were used in PCA and PLSR. Data in PCA and PLSR were centered, full crossvalidated, and standardized. Sensory data and data on chemical and physical properties of the pastes were used as explanatory variables (X matrix) while means of overall consumer acceptance data were used as response variables (Y matrix) (Helgesen et al. 1997; Resano et al. 2010).

To identify consumer subgroups sharing common preference patterns, hierarchical cluster analysis using complete linkage and squared Euclidian distance was performed on consumer overall acceptance data. Means of overall acceptance obtained for each cluster and data on sensory, chemical, and physical properties of the pastes were used to obtain a PREFMAP of the clusters. The sensory, chemical, and physical properties data provided the X matrix while means of overall acceptance of clusters provided the Y matrix. Demographic information of the subgroups obtained through cross-tabulations provided an understanding of cluster compositions.

ANOVA, cluster analysis, cross-tabulations, and correlations were performed in SPSS 15.0 (SPSS Inc., Chicago, IL) while PCA and PLSR were performed in UnscramblerX 10.2 (CAMO Software, AS, Norway).

## Results and Discussion

### Chemical and physical properties of the pastes

There were significant differences (*P* < 0.05) in pH of the samples between the NFP and the LFP. LAB-fermented pastes had lower pH values ranging from 3.91 to 4.26 compared to NFP that had pH values ranging from 5.36 to 5.81 (Table 2). There was an agreement between lactic acid content, presented as titratable acidity, and pH levels in the pastes and the sensory perception of sourness. Lactic acid contents were higher in LFP than in NFP and so were the perceived sourness intensities. On the contrary, the amino acid contents did not agree with umami, bitterness, and sweetness taste perceptions. Amino acids in their free state (as L, D, and DL) contribute to bitter, sweet, and umami tastes in most foods. In this study, amino acids responsible for bitterness and umami were generally high in NFP while those responsible for sweetness were high in LFP (Table 2). However, perceived intensities of these tastes by descriptive sensory panel (Table 3) differed from the expectation from the chemical analyses. Panelists rated LFP high in bitterness and umami while NFP were rated high in sweetness. Descriptive sensory perception of bitterness was high in LFP probably because of interactions of the bitter compounds and the other tastants in the fermented pastes. According to Mukai et al. (2007), mixtures of bitter and sweet tastes resulted in variable effects at low intensity/concentration, while mixtures at moderate and high intensity/concentrations were mutually suppressive. In LFP, mixtures of sweet and bitter tastes were at low concentrations resulting in enhancement of bitter taste. While in NFP, the concentrations of sweet tastes were moderate and the overall concentrations of bitter tastes were high, resulting in suppression of bitterness. Furthermore, bitterness in LFP could have been enhanced due to interactions between sour and bitter compounds at low concentrations (Mukai et al. 2007). On the other hand, bitterness in NFP could have been reduced by aspartic and glutamic acids. Although there were no significant differences in aspartic acid contents among all samples, 90S had the highest content. Furthermore, glutamic acid content was highest in 100S and the content was significantly different between 100S and the rest of the pastes except 90S (Table 2). Thus overall, the amino acids imparting umami flavor were higher in NFP. Aspartic and glutamic acids were reported to be effective in reducing bitterness of solutions comprising bitter amino acids in low concentrations (Lindqvist 2010). Apart from amino acids, bitterness in soybeans is also influenced by bitter isoflavone glucosides, which are hydrolyzed during fermentation to bitter isoflavone aglycones (Drewnowski and Gomez-Carneros 2000). Salt content ranged from 228 to 272 mg/L (0.037–0.046%) and was low compared to other fermented soybean pastes, which can contain up to 14% salt (Kim et al. 2010). Salt was mainly due to chlorides naturally present in plants. Although saltiness was rated high in LFP, there were no significant differences (*P* > 0.05) in salinity among the samples. This study agrees with the suggestion that the interaction between tastes is not a fixed action depending on the intensity/concentration of each taste, but rather an enhancing or inhibitory effect, changing with the combined pattern of intensity and concentration (Mukai et al. 2007).

**Table 2:** Physical and chemical analyses of the fermented pastes.

Parameter^1^	Taste	100S	100SBS	90S	90SBS	75S	75SBS
pH		5.81 ± 0.59^a^	4.26 ± 0.28^c^	5.36 ± 0.14^b^	4.01 ± 0.31^c^	5.41 ± 0.18^b^	3.91 ± 0.29^c^
Titratable acidity (TA)	Sourness	0.58 ± 0.31^a^	0.56 ± 0.13^a^	0.37 ± 0.08^b^	0.68 ± 0.16^ac^	0.50 ± 0.18^a^	0.85 ± 0.24^c^
Histidine (His)	Bitterness	0.38	n.d	0.24	n.d	0.07 ± 0.05	n.d
Arginine (Arg)	Bitterness	0.06^ab^	0.07 ± 0.01^ab^	0.07 ± 0.06^ab^	0.05 ± 0.01^ab^	0.04 ± 0.02^a^	0.10 ± 0.03^b^
Tyrosine (Tyr)	Bitterness	0.07 ± 0.05^ab^	0.06 ± 0.02^ab^	0.18 ± 0.19^a^	0.04 ± 0.01^b^	0.08 ± 0.06^ab^	0.04 ± 0.02^b^
Valine (Val)	Bitterness	1.00 ± 0.31^a^	0.53 ± 0.21^ab^	0.89 ± 0.95^a^	0.26 ± 0.14^b^	0.50 ± 0.35^ab^	0.18 ± 0.10^b^
Methionine (Met)	Bitterness	0.03 ± 0.02^a^	0.04 ± 0.02^a^	0.05^a^	0.02^a^	n.d	0.01^a^
Isoleucine (Iso)	Bitterness	0.55 ± 0.16^ac^	0.13 ± 0.06^a^	0.65 ± 0.72^c^	0.04 ± 0.03^b^	0.32 ± 0.21^ac^	0.1 ± 0.07^a^
Phenylalanine (Phe)	Bitterness	2.19 ± 0.81^ab^	0.96 ± 0.71^ab^	2.59 ± 2.85^b^	0.42 ± 0.40^a^	1.11 ± 0.77^ab^	0.28 ± 0.27^a^
Leucine (Leu)	Bitterness	1.66 ± 0.91^a^	0.38 ± 0.20^b^	1.61 ± 1.76^a^	0.22 ± 0.12^c^	0.67 ± 0.58^ab^	0.17 ± 0.10^c^
Aspartate (Asp)	Umami	0.79 ± 0.40^a^	0.78 ± 0.10^a^	1.23 ± 0.86^a^	0.90 ± 0.03^a^	0.78 ± 0.31^a^	0.72 ± 0.12^a^
Glutamate (Glu)	Umami	4.84 ± 0.39^a^	2.55 ± 0.31^b^	3.71 ± 2.16^ab^	3.07 ± 0.26^b^	2.38 ± 0.59^b^	3.14 ± 1.06^b^
Serine (Ser)	Sweetness	0.63 ± 0.06^a^	0.18 ± 0.04^b^	0.29 ± 0.25^b^	0.19 ± 0.01^b^	0.3 ± 0.14^b^	0.18 ± 0.01^b^
Glycine (Gly)	Sweetness	0.47 ± 0.08^ac^	1.07 ± 0.38^b^	0.57 ± 0.25^ac^	1.06 ± 0.05^b^	0.29 ± 0.13^c^	0.73 ± 0.01^a^
Alanine (Ala)	Sweetness	3.63 ± 0.38^a^	3.84 ± 1.25^a^	2.63 ± 1.90^ab^	3.27 ± 0.54^a^	1.43 ± 0.34^b^	2.54 ± 0.66^a^
Lysine (Lys)	Sweetness	0.95 ± 0.06^abc^	1.49 ± 0.63^a^	0.92 ± 0.53b^c^	1.47 ± 0.21^ab^	0.74 ± 0.24^c^	0.78 ± 0.19^c^
Salinity	Saltiness	240 ± 32.66^a^	262.5 ± 23.63^a^	228.75 ± 49.39^a^	241.25 ± 33.26^a^	245 ± 19.15^a^	272.5 ± 22.55^a^

Means not sharing a superscript within a row are significantly different (*P* < 0.05). Samples coded 100S, 90S, and 75S represent naturally fermented pastes, while samples coded 100SBS, 90SBS, and 75SBS represent lactic acid-fermented pastes. Pastes are designated according to 100%, 90%, and 75% soybean composition, the remaining proportions being maize.

1 Units of measurement: titratable acidity (g lactic acid/100 g sample), amino acids (*μ*mol/g), salinity (mg/L).

**Table 3:** Mean intensity scores, *F* ratios and *P*-values of descriptors/attributes based on product effect.

Attribute	100S	100SBS	90S	90SBS	75S	75SBS	*F* ratio	*P*-value
Raw soybean odor	7.17 ± 3.52^a^	4.37 ± 1.65^bc^	5.07 ± 2.35^ab^	3.43 ± 1.65 ^cd^	6.00 ± 2.84^ab^	2.80 ± 1.73^d^	7.51	<0.000
Roasted soybean aroma	4.21 ± 1.65^a^	5.1 ± 1.90^b^	4.67 ± 2.31^ab^	4.53 ± 2.85^ab^	4.4 ± 2.90	4.40 ± 2.55a	2.67	0.03
Burnt roasted soybean odor	2.55 ± 1.15^a^	4.9 ± 2.23^b^	3.67 ± 2.22^c^	4.93 ± 1.99^b^	3 ± 1.51^ac^	5.10 ± 2.34^b^	10.15	<0.000
Roasted maize aroma	3.2 ± 1.63^a^	4.6 ± 1.96^b^	4.21 ± 2.04^ab^	3.33 ± 1.45^a^	4.33 ± 3.03^b^	3.87 ± 1.78^ab^	2.12	0.07
Burnt roasted maize odor	2.7 ± 1.68^a^	4.57 ± 2.37^b^	3.43 ± 2.25^ac^	4.03 ± 1.87^bc^	3.4 ± 2.65^ac^	4.43 ± 2.16^b^	3.80	0.00
Soaked burnt roasted maize odor	3.07 ± 1.99^ab^	3.53 ± 2.92^a^	3.55 ± 2.72^a^	3.13 ± 2.33^ac^	2.37 ± 1.47^bc^	4.00 ± 3.04^a^	2.41	0.04
*Chigumuyoyo* aroma	2.13 ± 1.36^a^	4.17 ± 2.33^b^	3.33 ± 1.99^c^	4.47 ± 2.42^b^	2.83 ± 1.84^ac^	5.67 ± 2.42^d^	8.62	<0.000
*Chitumbuwa* aroma	1.9 ± 1.03^a^	4.87 ± 2.74^b^	3.53 ± 2.37^c^	9.07 ± 2.75^d^	2.83 ± 1.86^ac^	9.44 ± 3.19^e^	48.82	<0.000
*Mandazi* aroma	3.4 ± 1.48^ac^	3.57 ± 2.03^ab^	3 ± 1.58^a^	4.55 ± 2.38^b^	3.9 ± 2.07^bc^	6.73 ± 3.05^d^	12.54	<0.000
Fermented aroma	6.93 ± 3.66^a^	4.07 ± 2.08^b^	4.2 ± 2.12^b^	5.63 ± 2.76^c^	4.87 ± 3.09^bc^	4.53 ± 2.37^bc^	5.37	0.00
*Matsukwa* odor	3.2 ± 1.92^a^	3.97 ± 1.94^a^	3.37 ± 1.94^a^	3.4 ± 2.21^a^	3.13 ± 2.01^a^	3.20 ± 2.37^a^	0.44	0.83
*Kondoole* aroma	3.8 ± 2.66^a^	3.43 ± 1.89^ab^	2.87 ± 1.63^bc^	2.8 ± 1.81^c^	2.9 ± 2.01^ac^	2.66 ± 1.80^bc^	1.26	0.29
*Thobwa* aroma	4.10 ± 2.55^a^	3.71 ± 2.53^ab^	2.71 ± 1.65^c^	3.33 ± 2.18^ac^	2.91 ± 1.81^bc^	3.35 ± 2.35^bc^	2.55	0.03
*Chambiko* aroma	2.73 ± 1.57^a^	2.97 ± 1.87^a^	2.7 ± 1.91^a^	2.73 ± 2.24^a^	2.63 ± 1.94^a^	2.77 ± 2.15^a^	0.37	0.87
Fermented beans aroma	2.57 ± 1.55^ab^	2.73 ± 2.02^b^	2.27 ± 1.51^a^	2.6 ± 2.04^ab^	1.8 ± 1.03^a^	2.3 ± 1.78^ab^	1.18	0.33
Fried egg aroma	3.43 ± 1.91^a^	3.2 ± 1.83^a^	3.13 ± 1.53^a^	2.5 ± 1.59^b^	3.67 ± 2.43^a^	2.2 ± 1.38^b^	4.12	0.00
*Mafuta a chiwisi* odor	2.57 ± 2.27^a^	2.5 ± 2.10^a^	2.5 ± 1.94^a^	2.43 ± 1.92^a^	2.33 ± 2.26^a^	2.67 ± 2.37^a^	0.10	0.99
Rancid odor	9.07 ± 4.02^a^	4.87 ± 2.36^bcd^	5.07 ± 2.42^c^	3.4 ± 2.16^d^	5.8 ± 2.46^c^	3.57 ± 2.22^bd^	13.37	<0.000
Brown	2.83 ± 2.10^a^	7.2 ± 3.03^b^	3.07 ± 2.09^a^	11.03 ± 2.40^c^	2.8 ± 1.86^a^	12.97 ± 1.40^d^	101.65	<0.000
Yellow	10.07 ± 2.82^a^	5.5 ± 2.95^b^	9.8 ± 2.59^a^	2.5 ± 1.31^c^	10.6 ± 2.40^d^	1.33 ± 0.55^c^	74.90	<0.000
*Chitumbuwa*-like	3.37 ± 2.95^a^	6.43 ± 2.25^b^	3.7 ± 2.55^a^	8.35 ± 2.98^c^	2.7 ± 1.56^d^	8 ± 3.62^c^	23.78	<0.000
*Mandazi*-like	2.67 ± 2.16^ac^	5.23 ± 2.43^b^	3.47 ± 2.36^c^	6.97 ± 3.30^bd^	2.57 ± 1.72^a^	7.63 ± 2.79^d^	19.36	<0.000
Fried egg-like	7.73 ± 2.74^a^	4.37 ± 2.55^b^	7.5 ± 2.70^a^	1.69 ± 0.81^c^	7.73 ± 2.84^a^	1.3 ± 0.60^c^	42.25	<0.000
Sweetness	2.07 ± 1.36^ac^	1.67 ± 0.71^b^	1.83 ± 0.83^bc^	1.4 ± 0.50^b^	2.63 ± 1.97^a^	1.7 ± 0.65^b^	4.77	0.001
Saltiness	1.93 ± 0.91^acd^	2.53 ± 1.91^ab^	1.57 ± 0.77^c^	2.63 ± 2.55^a^	1.83 ± 0.95 ^cd^	2.7 ± 1.97^ad^	3.39	0.007
Umami	3.5 ± 1.72^a^	5.23 ± 2.58^b^	3.5 ± 1.81^a^	6.07 ± 2.86^b^	3.47 ± 1.80^a^	7 ± 3.09^c^	17.29	<0.000
Sourness	3 ± 1.49^a^	7.08 ± 3.05^b^	5.47 ± 2.85^c^	10.07 ± 3.29^d^	4.4 ± 2.28^c^	10.9 ± 3.24^d^	32.17	<0.000
Bitterness	1.8 ± 1.49^ad^	2.48 ± 2.38^ab^	1.6 ± 1.16^ac^	2.9 ± 2.86^b^	1.47 ± 0.78 ^cd^	3.0 ± 3.49^b^	3.11	0.01
Aftertaste	3.13 ± 2.16^ac^	4.27 ± 2.42^ab^	2.6 ± 1.59^c^	4.6 ± 2.96^ab^	2.53 ± 1.80^c^	5.1 ± 2.68^b^	5.09	0.000
Surface roughness	5.2 ± 2.55^a^	6.07 ± 2.83^a^	5.7 ± 2.44^a^	7.5 ± 3.15^b^	7.73 ± 2.52^b^	8.4 ± 2.94^b^	6.01	<0.000
Softness	7.63 ± 3.46^a^	6.6 ± 3.29^ab^	5.93 ± 3.26^a^	5.03 ± 3.40^c^	5.17 ± 2.89^bc^	4.03 ± 2.76^c^	7.08	<0.0001
Easiness to break	6.47 ± 2.97^a^	6.97 ± 2.91^a^	6.83 ± 2.65^a^	7.5 ± 4.02^a^	6.37 ± 3.05^a^	6.77 ± 3.38^a^	0.58	0.72
Graininess	3.9 ± 1.90^a^	4.35 ± 2.35^a^	4.5 ± 2.42^a^	4.53 ± 2.30^a^	6.17 ± 2.96^b^	4.97 ± 3.09^a^	2.89	0.02
Sogginess	8.5 ± 2.55^a^	7.11 ± 3.05^a^	6.31 ± 2.52^b^	8.6 ± 2.81^a^	5.31 ± 2.42^b^	11.12 ± 2.85^c^	20.79	<0.000

Intensity based on a scale of 1–15 (1 = none or least or very weak, 15 = very strong intensity). Means not sharing a superscript within a row are significantly different (*P* < 0.05). Samples coded 100S, 90S, and 75S represent naturally fermented pastes, while samples coded 100SBS, 90SBS, and 75SBS represent lactic acid-fermented pastes. Pastes are designated according to 100%, 90%, and 75% soybean composition, the remaining proportions being maize. Full names of terms and their meanings are given in Table 1.

All the samples behaved as viscoelastic solids. Tests in normal rotation were not done as the samples slipped on the rheometer surfaces before yield occurred. The reason for the slimy sample surface was probably due to the presence of exopolysaccharides produced by some LAB. There were no significant differences in relative stiffness between NFP and LFP.

### Descriptive sensory analysis

Thirty-four descriptors/attributes describing appearance, aroma/odor, taste, and texture were generated to characterize the sensory properties of the fermented pastes (Table 1). There was high agreement among panelists in rating the intensities of the attributes as observed from the profile plots (data not shown) as most assessor lines followed the consensus lines closely (Tomic et al. 2010). Out of the 34 attributes, 27 were significantly different (*P* < 0.05) on product effect. The attributes not significantly different were roasted maize, *kondoole*,*chambiko*, and fermented beans aromas, *mafuta a chiwisi* odor, and readiness to be broken (Table 1 presents meanings of descriptors). Only attributes that were significantly different on product effect were used in further analyses.

Differences among samples in the following attributes: burnt roasted soybean, *chitumbuwa* and *mandazi* aromas, rancid odor, brown and yellow colors, *chitumbuwa-*like, *mandazi-*like, and fried egg-like appearances, umami, and sourness tastes and soggy texture were clearly discriminated by panelists as observed from the high *F* ratios (Table 3). Overall, the panel's ability to discriminate between samples was good, although Tucker plots (data not shown) showed that some assessors had low discrimination ability in a few attributes, namely graininess, roasted soybean, soaked roasted maize and *thobwa* aromas. These attributes had relatively low *F* ratios as well (Table 3).

Significant correlations were observed among sensory descriptors. Attributes strong in intensities in NFP had significant (*P* < 0.001) positive correlations with each other and this trend were similar in LFP (Table 4). Conversely, attributes strong in intensities in NFP negatively correlated with those strong in intensities in LFP. Strong correlations were observed amongst appearance attributes (*r* = −0.439 to −0.844). Brown color strongly positively correlated with *chitumbuwa-like* and *mandazi-like* appearances and also negatively correlated with yellow color and fried egg-like appearance. Appearance attributes also strongly correlated with aroma attributes and some tastes. For instance, brown color, *chitumbuwa-like*, and *mandazi-like* appearances showed significant (*P* < 0.001) positive correlations with *chitumbuwa* aroma, umami, bitterness, aftertaste, and sourness. *Mandazi* aroma positively correlated with *thobwa* aroma, fried egg aroma, umami, and aftertaste. Egg-like appearance positively correlated with sweetness. The intensities of attributes with significant positive correlations with brown color were high in LFP, while the intensities of attributes with significant positive correlations with yellow color were high in NFP. Therefore, the type of fermentation greatly influenced the appearance of the fermented pastes. Among aromas that strongly correlated with each other were fermented aroma and rancidity. Fermented aroma intensity and rancidity were highest in 100S; in addition, rancidity was high in all NFP (Table 3). High fermented aroma intensity in 100S could be due to uneven fermentation in NFP due to spontaneous fermentation by natural microflora over a long period (Kim et al. 2010). Significant positive correlations were also observed between textural properties, including roughness and graininess, which were attributes influenced by composition.

**Table 4:** Pearson correlations between descriptors/attributes characterizing the fermented pastes.

	RawS	BRoastdS	Chigumu	ChituA	MandA	Rancid	Brown	Yellow	ChituL	MandL	EggL	Umami	Thobwa
Brown	−0.468	0.477	0.358	0.665	0.462	−0.437	1						
Yellow	0.470	−0.391	−0.394	−0.670	−0.350	0.367	−0.844	1					
ChituL	−0.356	0.496		0.503	0.339	−0.256	0.736	−0.617	1				0.371
MandL	−0.344	0.410	0.271	0.526	0.448	−0.375	0.733	−0.563	0.672	1			0.327
EggL	0.334	−0.290	−0.299	−0.612	−0.315	0.363	−0.717	0.757	−0.444	−0.439	1		
Umami	−0.280	0.303		0.409	0.268	−0.271	0.496	−0.441	0.555	0.497	−0.374	1	
Sour	−0.366		0.527	0.719	0.219	−0.292	0.577	−0.643	0.355	0.411	−0.603	0.365	
AfterT		0.347			0.326		0.446	−0.351	0.405	0.376	−0.247	0.361	0.369
Soggy			0.353	0.491			0.362	−0.383	0.226	0.292	−0.360	0.251	
MandA	−0.231			0.297									0.404
Rancid	0.412			−0.277									
Sweet				−0.296			−0.224				0.283		
Bitter		0.248			0.224		0.336	−0.262	0.298				0.294
Rough					0.283	−0.274	0.262	−0.238	0.261			0.296	
Soft	0.221			−0.287		0.259		0.256					0.284
Salty			0.372	0.261			0.267	−0.252			−0.237		
Grainy		0.269			0.268								0.360
ChituA	−0.362	0.381	0.573										
SBRoastM		0.291			0.266				0.292	0.335			0.553
BRoastM		0.509		0.263	0.381		0.415		0.353	0.393	−0.230		0.337
	RoastS		BRoastM		Sour		EggA		Grainy	Bitter	SBRoastM		FermA
BRoastS	0.476	SBRoastM	0.335	Soft	−0.261	EggL	0.327	AfterT	0.258	0.375	0.229	RawS	0.347
BRoastM	0.346	Salty	0.244	Soggy	0.472	MandA	0.266	Rough	0.245			Rancid	0.574
ChituL	0.280	Grainy	0.295	Sweet	−0.277	Grainy	0.282	SBRoastM	0.219	0.259	1		
Thobwa	0.357			Rough	0.235	SBRoastM	0.251	Salty		0.238			
Grainy	0.270					AfetrT	0.282						
	Rough		Soggy			Sweet	0.270						
Soft	−0.228	Sweet	−0.280										

Only attributes showing significant correlations at *P* = 0.001 are presented. Full names of terms and their meanings are given in Table 1.

#### Sensory properties of the fermented pastes

**Figure 1:**
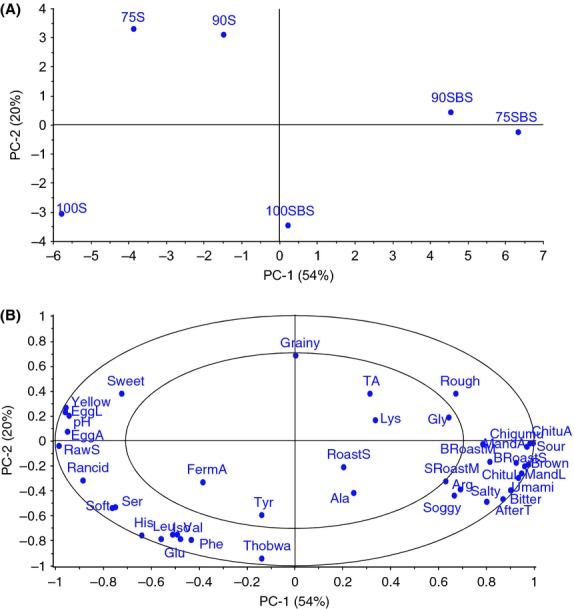
Principal component analysis of fermented pastes and sensory attributes. (A) Score plot showing relatedness of samples in terms of sensory, chemical, and physical properties of the pastes. (B) Correlations loading plot showing sensory properties of the pastes. On the map, 100S, 90S, and 75S represent naturally fermented pastes (NFP) while 100SBS, 90SBS, and 75SBS represent lactic acid-fermented pastes (LFP). Pastes are designated according to 100%, 90%, and 75% soybean composition, the remaining proportions being maize.

Sensory properties characterizing the products are shown in PCA map in Figure 1. The first two principal components (PC1 and PC2) explained 74% of the variation. This highly explained variance in PC1 and PC2 shows that there was high systematic variation within the data, indicating that the panel discriminated well between the products. The score plot (Fig. 1A) shows product distribution in multivariate space and PC1 explains differences in the products according to type of fermentation, distinguishing NFP on the left from LFP on the right. Attributes responsible for this categorization were appearance, some odors/aromas, taste, and pH. The mean intensities of yellow color, fried egg-like appearance, raw soybean odor, fried egg aroma, and pH were high in NFP. These attributes also loaded highly on the negative side of PC1 (Fig. 1B) and were sensory properties characterizing NFP. On the positive side of PC1, brown color, *chitumbuwa-* and *mandazi*-like appearances, burnt roasted soybean odor, *chigumuyoyo* and *chitumbuwa* aromas, umami, bitterness, aftertaste, and sourness loaded highly (Fig. 1B). These attributes had high intensities in LFP, hence characterized LFP. Most of the amino acids responsible for bitterness (histidine, arginine, tyrosine, valine, isoleucine, phenylalanine, leucine) and glutamate responsible for umami loaded highly on the negative side of PC1. Due to the comparatively higher content of bitter amino acids, it would be expected that NFP would have higher bitterness intensity compared to LFP. On the contrary, LFP were perceived to be more bitter than NFP, probably because of taste interactions in which glutamate could have suppressed bitterness in NFP and sourness could have enhanced bitterness in LFP.

The proximity of 90S and 75S; and 90SBS and 75SBS on the PCA map (Fig. 1A) indicates close similarity in terms of sensory properties, unlike 100S and 100SBS, which are clearly separated from the other NFP and LFP, respectively. This delineation is along PC2 on which graininess and sweetness loaded highly on the positive dimension, while *thobwa* aroma, fermented aroma, and softness loaded highly on the negative dimension (Fig. 1B). The intensities of fermented aroma and softness were high in 100S, while the intensities of sweetness and graininess were high in 90S and 75S.

Yellow color of NFP originated from the color of soybeans, while brown color in LFP could be due to caramelization and Maillard reactions. Color of fermented soybean pastes like *doenjang/miso* is due to the raw materials used, amino carbonyl reaction of Maillard browning, oxidative browning, enzymatic browning, and browning enhancers (Chung and Chung 2007). In this study, 100S, 90S, and 75S underwent natural fermentation, while 100SBS, 90SBS, and 75SBS were BS with a LAB-fermented product; hence, type of fermentation had a major influence on color, giving the strong browning intensity in LFP. LAB fermentations increase the amount of reducing sugars in products (Sripriya et al. 1997) and the sugars could be responsible for the browning intensity due to caramelization and participation in Maillard reactions with amino carbonyls during frying. As increasing pH values enhance both caremelization and Maillard reactions (Ajandouz and Puigserver 1999; Ajandouz et al. 2001), it would be expected that NFP would have stronger browning intensities than LFP. However, all the samples had pH values below 6.0, thus were slightly acidic and offered some stability of the amino acids that were heated in the presence of reducing sugars (Ajandouz and Puigserver 1999). The difference in browning intensity was probably due to the difference in the amount of reducing sugars, which were more in LFP than in NFP (data not shown).

Tastes of the pastes were significantly different (*P* < 0.05). NFP had low sourness intensity and their pH values were only slightly reduced from the initial. However, sweetness intensities were higher in NFP and particularly in 75S, probably because of its high maize content; hence high content of sugars resulted in higher sweet intensity. On the other hand, LFP were positively associated with sourness, umami, bitterness, saltiness, and aftertaste. The intensities of these tastes were highest in 75SBS. Chung and Chung (2007) found that fermented soybean products with high saltiness also had high umami (monosodium glutamate, MSG) and sour tastes. Although salt was not added to all the samples, its perception could be due to the presence of NaCl and KCl, which were attributed to saltiness perception in Korean-fermented soybean pastes, *doenjang* (Kim and Lee 2003). Besides, salt content alone does not sufficiently predict perceived saltiness intensity as synergistic interactions of salt and other flavor compounds also affect saltiness perception (Kim et al. 2010). Sourness in fermented products is due to organic acids, which increase during LAB fermentations. Because 75SBS had the highest maize content, it provided more fermentable sugars as substrate for organic acid production by LAB. In soybean-fermented pastes, malic, citric, succinic, and lactic acids are responsible for the sour taste (Kim et al. 2010). In this study, sour taste could have been due to succinic, lactic, and acetic acids, which were detected (data not shown). Umami taste is related to glutamic and aspartic acids (Kim and Lee 2003), which are present in soybeans and tend to increase with fermentation (Dajanta et al. 2011). Another study on similar products (Kim et al. 2010) reported high bitterness intensities, which were attributed to bitter amino acids produced during fermentation. Amino acids responsible for bitter taste include leucine and isoleucine (Namgung et al. 2010). Salts, sugars, organic acids, umami compounds, amino acids, Maillard peptides, types of base ingredients, microorganisms, and various aroma compounds contribute to flavors of fermented soybean products (Chung and Chung 2007).

A range of aromas and odors were described. NFP had high intensities of raw soybean odor, rancid odor, fermented aroma, and fried egg aroma. Raw soybean and rancid odors are among the flavors that reduce consumer acceptance of soy products (Torres-Penaranda et al. 1998). The two odors were highest in 100S. On the other hand, LFP were characterized by aromas associated with roasted soybeans and maize. In this case, LAB fermentation was able to mask the characteristic beany and rancid odors of soybeans that are due to oxidation of polyunsaturated lipids catalyzed by lipoxygenase (Ediriweera et al. 1987). Rancid odor is associated with volatile compounds such as 3-methylbutanoic acid, 2-methylpropanoic acid, and butanoic acid, a major compound in different fermented soybean foods (Jo et al. 2011).

Textural differences were also described. 100S was rated softer than the rest of the samples while graininess and roughness intensities were high in products containing maize, particularly 75S, 75SBS, and 90SBS. Differences in composition of the products accounted for the differences in textural properties, resulting in rough appearance and large particle sizes in the products containing maize. Additionally, LFP absorbed more oil during frying than NFP; this tendency could have been due to their slightly higher moisture content (data not shown), which led to more oil uptake as the water evaporated during frying (Krokida et al. 2000).

### Consumer acceptance

A total of 150 consumers participated in the consumer acceptance study but demographic information was collected on 148. A consumer was defined as a person who occasionally consumed soybeans and soybean-based products. At the time of the study, 32.4% of the participants had consumed soybean-based products within the past 2 months from the date of data collection. Soybeans were mostly consumed in porridge (69%), although some of the consumers used texturized soy products locally known as *soya pieces* as relish (side dish), roasted beans as snack, soy flour as a condiment in vegetables and other foods, in addition to using soybeans in porridge (Table 5). Soybean flour is used together with maize flour in a weaning food prepared as porridge and locally known as *Likuni Phala*. Most consumers (88%) were aware that soybeans are nutritious as they associated the promotion of its use in growth-monitoring centers and in nutrition rehabilitation programs for under-5 children.

**Table 5:** Ways of household soybean consumption by the consumers.

Ways of consumption	Consumers (%) *n* = 129
Porridge only	89 (69)
Porridge and soya pieces	8 (6.2)
Soya pieces only	11 (8.5)
Porridge and roasted soybeans as snack	7 (5.4)
Porridge and soy flour vegetable condiment	8 (6.2)
Porridge, roasted soybeans, and soya pieces	4 (3.1)
Used in maize flour-based snacks	2 (1.6)

#### External PREFMAP

**Figure 2:**
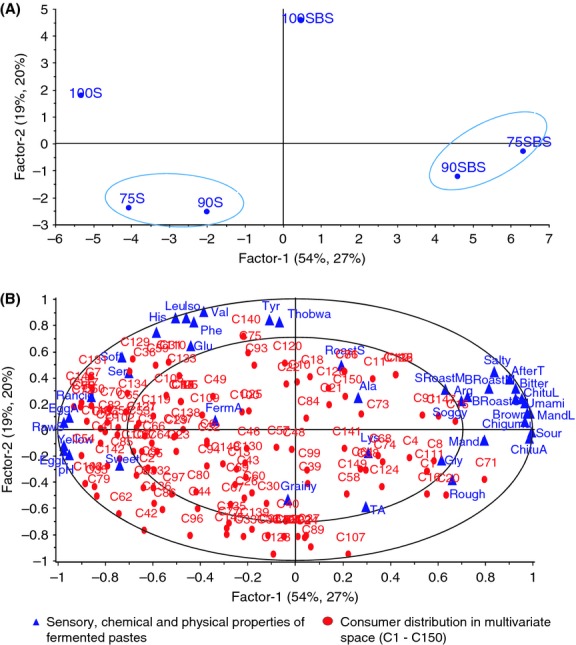
External preference mapping showing sensory attributes driving consumer preference of the pastes. (A) Grouping of pastes according to sensory, chemical, and physical properties and consumer preference. (B) Consumer preference pattern as influenced by sensory, chemical, and physical properties. On the map, 100S, 90S, and 75S represent naturally fermented pastes, while 100SBS, 90SBS, and 75SBS represent lactic acid-fermented pastes. Pastes are designated according to 100%, 90%, and 75% soybean composition, the remaining proportions being maize.

To understand the attributes driving consumer liking, sensory, chemical, and physical data were regressed with consumer acceptance data using PLSR. The sensory, chemical, and physical data were used as predictor variables, while overall consumer acceptance data were used as response variables. In Figure 2, PC1 and PC2 together explain 73% of the variation in the pastes in terms of their properties and 47% of the variation in consumer preference for the pastes. The location of the samples on the map is based mainly on sensory attributes and the preference pattern shows that consumers also used the same underlying sensory properties to make their acceptability. The high density of consumers in the two left quadrants (Fig. 2B) indicates that the direction of preference was toward NFP. These samples were characterized by strong yellow color, higher pH, fried egg-like appearance, and aroma, sweetness, softness, rancid odor, and raw soybean odor. It appears that the positive impact of higher pH (low sourness intensity), sweetness, and fried egg aroma exceeded the negative impact of rancid and raw soybean odors. These two odors have been documented as deterring consumer acceptance of soybeans (Gupta 1997; Torres-Penaranda et al. 1998). Therefore, higher pH (low sourness intensity), fried egg aroma, and sweet taste seem to be the drivers of liking of the fermented soybean pastes, especially for NFP for most consumers. Nevertheless, other consumers preferred LAB-fermented pastes, which had strong brown color, sourness, bitterness, saltiness, umami, burnt roasted soybeans, and maize aromas. These attributes seem to be drivers of liking of 90SBS and 75SBS and as they load directly opposite drivers of liking of NFP, they can be considered as drivers of disliking for consumers preferring 75S, 90S, and 100S.

Taste and pleasure are among the most important predictors of food choice (Brunsø et al. 2002). Bitterness and strong sourness could have been the key attributes leading to little acceptance of LFP. Bitter taste is a major problem in the food and pharmaceutical industries due to its negative hedonic impact on ingestion (Drewnowski and Gomez-Carneros 2000; Ley 2008). In most cases, the bitter taste is not desirable and has to be eliminated from or masked in the product to increase a product's acceptance. Umami is the savory delicious taste in meat, poultry, sea foods, and fermented beans (Yamaguchi and Ninomiya 2000). Although umami is among the drivers of dislike in this study, it could not have been the reason for dislike of LFP. Its inclusion among drivers of disliking in this study is because of its significant (*P* < 0.001) correlation with sourness and aftertaste (*r* = 0.365 and 0.361, respectively) intensities. Consumption of foods that are strong in sourness is typically avoided (Breslin and Spector 2008).

In this study, mixed strains of LAB from *thobwa* were used in the fermentation of LFP. To improve acceptance of LAB-fermented soybeans, selection of strains that results in desirable sensory properties would be recommended. This strain selection can be achieved through identification and characterization of LAB involved in soybean fermentation.

#### Consumer preferences according to clusters

**Figure 3:**
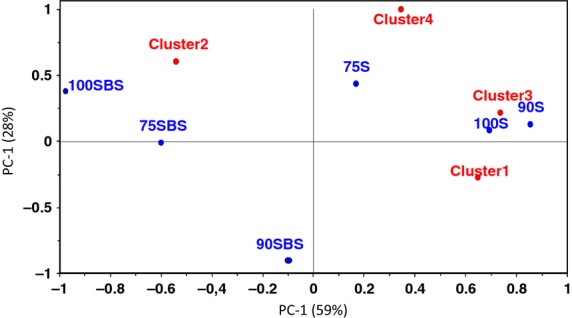
Principal component analysis of fermented pastes and consumer clusters. Naturally fermented pastes (100S, 90S, 75S) and lactic acid bacteria-fermented pastes (100SBS, 90SBS, and 75SBS). Pastes are designated according to 100%, 90%, and 75% soybean composition, the remaining proportions being maize.

A visual inspection of the PREFMAP (Fig. 2) reveals heterogeneity in consumers' acceptability, although more consumers liked the NFP. Figure 2A shows four clusters as follows: 100S; 90S and 75S; 75SBS and 90SBS; and 100SBS. These clusters are mainly based on sensory properties as the PREFMAP is based on the PCA representation of sensory attributes (Resano et al. 2010). To understand this heterogeneity in consumer preference pattern more, a cluster analysis using a 6 × 150 matrix of pastes and overall consumer acceptance scores was performed. Cluster analysis assigned consumers with similar preference patterns to one group resulting in four clusters as well. A PCA of mean overall acceptance of the clusters and the pastes was then obtained (Fig. 3). The clustering pattern was slightly different from the pattern in Figure 2. Clusters 1 and 3 were composed of consumers who liked 100S and 90S, cluster 2 was composed of consumers who preferred LFP with a bias of 100SBS and 75SBS, while cluster 4 was composed of consumers that liked 75S. As seen in Figure 2, the direction of preference is biased toward NFP. The composition of consumers in each cluster is shown in Table 6. Consumers in cluster 2 disliked NFP and preferred LFP, particularly 100SBS. Consumers in cluster 3 liked 90S and 100S and disliked all the sour LFP products and 75S, while consumers in cluster 4 liked 75S and were slightly tolerant of the sour products except for 90SBS (Table 7).

**Table 6:** Demographic information of the clusters (numbers in parentheses are percentages).

Demography	Cluster 1	Cluster 2	Cluster 3	Cluster 4	Total
Sex					
Male	22 (14.9)	3 (2.0)	2 (1.4)	4 (2.7)	31 (20.9)
Female	79 (53.4)	9 (6.1)	11 (7.4)	18 (12.2)	117 (79.1)
Total	101 (68.2)	12 (6.1)	13 (8.8)	22 (14.9)	148 (100)
Age					
14–29	53 (35.8)	5 (3.4)	5 (3.4)	16 (10.8)	79 (53.4)
30–49	28 (18.9)	5 (3.4)	4 (2.7)	4 (2.7)	41 (27.7)
50–80	20 (13.5)	2 (1.4)	4 (2.7)	2 (1.4)	28 (18.9)

**Table 7:** Mean overall acceptance scores for consumer clusters.

Cluster	100S	100SBS	90S	90SBS	75S	75SBS
1	6.25 ± 1.35^a^	5.20 ± 2.1^a^	6.23 ± 1.46^a^	6.23 ± 0.90^a^	6.39 ± 1.10^a^	5.51 ± 1.84^a^
2	2.67 ± 2.01^b^	6.25 ± 0.87^a^	2.75 ± 2.18^b^	3.17 ± 2.86^b^	6.50 ± 0.8^a^	5.50 ± 2.02^ac^
3	5.00 ± 2.35^c^	1.31 ± 0.75^b^	6.15 ± 1.68^a^	2.69 ± 2.39^bc^	4.38 ± 2.96^b^	2.77 ± 2.49^b^
4	6.46 ± 0.67^a^	5.36 ± 1.18^a^	6.32 ± 1.09^a^	2.00 ± 1.31^c^	6.00 ± 1.35^a^	4.18 ± 2.04^c^

Means not sharing a superscript within a column are significantly different (*P* < 0.05). 100S, 90S, and 75S represent naturally fermented pastes, while 100SBS, 90SBS, and 75SBS represent lactic acid-fermented pastes. Pastes are designated according to 100%, 90%, and 75% soybean composition, the remaining proportions being maize.

Although 100SBS was rated lowest with an overall acceptance score of 1.31 by consumers in cluster 3 (Table 7), the same sample was rated 6.25 by consumers in cluster 2. Differences in overall acceptances of the same sample by different clusters underscore consumer heterogeneity. Attributes characterizing 100SBS also characterized LFP. These attributes were also considered as drivers of disliking by many consumers. Although the sensory attributes characterizing LFP were not necessarily highest in 100SBS, this paste stood out in terms of *thobwa* and roasted soybean aromas. These aromas loaded highly on PC2 (Fig. 2) and distinguished 100SBS from the other LFP samples, which were mainly characterized by attributes loading highly on PC1. These findings agree with the concept that consumer perception is complex and multidimensional. Consumers respond not only to a certain sensory input but also to other inputs perceived simultaneously and also to physical perceptual interactions among inputs (Costell et al. 2010).

**Figure 4:**
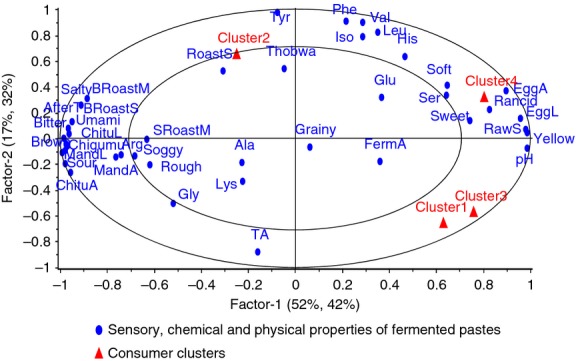
External preference mapping showing sensory attributes driving liking of the pastes by consumers in the clusters.

To understand attributes driving preference of consumers in these clusters, PLSR was performed with sensory, chemical, and physical data as X matrix and means for overall consumer acceptance of the clusters as Y matrix. Figure 4 shows sensory properties driving consumer liking in the clusters. Consumers in clusters 1, 3, and 4 had similar drivers of liking, with some drivers having a greater influence in some clusters than in others. For instance, clusters 1 (*n* = 101) and 3 (*n* = 13) were characterized by consumers who liked 100S and 90S. Drivers of liking of these products were yellow color, higher pH, raw soybean odor, and fermented aroma. In this case fermented aroma was the main driver. In addition to attributes driving liking in clusters 1 and 3, sweet taste, fried egg aroma, fried egg-like appearance, rancid odor, and soft texture were drivers of consumer liking in cluster 4 (*n* = 22). In cluster 2, the main driver of liking of consumers (*n* = 12) was roasted soybean aroma and *thobwa* aroma. Attributes that loaded highly on the opposite direction of attributes driving liking of the majority of the consumers can be considered as drivers of dislike of these products. Therefore, burnt roasted soybean odor, *chigumuyoyo* aroma, soaked burnt roasted maize aroma, *mandazi* aroma, *chitumbuwa* aroma, *mandazi-* and *chitumbuwa*-like appearances, sourness, bitterness, saltiness, aftertaste, and brown color were drivers of dislike for most consumers.

Cluster 1 was the largest in terms of consumer composition followed by cluster 4 (Table 6). There were no significant differences in overall acceptance of the products by consumers of cluster 1 (Table 7), even though liking was biased toward NFP. This indicates that both naturally fermented and LAB-fermented pastes have the potential of being used by the consumers. However, to increase utilization and acceptance of the fermented pastes, it would be necessary to optimize drivers of liking influencing acceptance of NFP. Thus, optimizing pH, softness, raw soybean odor, rancid odor, fermented aroma, sweet taste, fried egg aroma, and appearance, and yellow color by increasing the desirable properties while decreasing intensities of undesirable properties would increase acceptability and utilization of fermented pastes.

## Conclusions

The study concluded that the trained panel discriminated the products based on their type of fermentation; and consumers used similar discrimination in determining their preference patterns. Most consumers preferred NFP to LAB-fermented pastes. Strong intensities of yellow color, pH, sweet taste, raw soybean odor, rancid odor, fermented aroma, and soft texture in NFP were considered as positive. On the contrary, strong intensities of burnt roasted soybean odor, *chigumuyoyo* aroma, soaked burnt roasted maize odor, *mandazi* aroma, *chitumbuwa* aroma, *mandazi* and *chitumbuwa*-like appearances, sourness, bitterness, saltiness, aftertaste, and brown color, which characterized LFP were considered negative.

Consumer segmentation in liking of the products was identified, with direction of preference toward NFP. Consumers were assigned to four clusters, with the largest cluster composed of consumers who accepted all products almost similarly. This indicates that there is potential of utilization of both naturally and LAB-fermented soybean pastes. However, optimization either by increasing or reducing intensities of drivers of liking or disliking would be recommended to increase utilization of the fermented pastes. Because of heterogeneity, optimization of attributes which were the main drivers of liking in different clusters such as pH, raw soybean odor, rancid odor, soft texture, sweet taste, egg aroma, yellow color, egg-like appearance, fermented aroma, and roasted soybean aroma would be recommended. However, as pH values of NFP were relatively high, a food safety challenge is recognized for NFP.

Being the first study on fermented soybean and soybean/maize blend pastes in Malawi, the information provided could be used in future developments of similar products for wide acceptance and utilization of soybeans.

## References

[b1] Ajandouz EH, Puigserver A (1999). Nonenzymatic browning reaction of essential amino acids: effect of pH on caramelization and Maillard reaction kinetics. J. Agric. Food Chem.

[b2] Ajandouz EH, Tchiakpe LS, Ore FD, Benajiba A, Puigserver A (2001). Effects of pH on caramelization and Maillard reaction kinetics in fructose-lysine model systems. J. Food Sci.

[b3] Allagheny N, Obanu ZA, Campbell-Platt G, Owens JD (1996). Control of ammonia formation during *Bacillus subtilis* fermentation of legumes. Int. J. Food Microbiol.

[b4] Anderson RL, Wolf WJ (1995). Overview of soybean processing and products: compositional changes in trypsin inhibitors, phytic acid, saponins and isoftavones related to soybean processing. J. Nutr.

[b5] AOAC (1990). Official methods of analysis.

[b6] Asgar MA, Fazilah A, Huda N, Bhat R, Karim AA (2010). Nonmeat protein alternatives as meat extenders and meat analogs. Compr. Rev. Food Sci. Food Saf.

[b7] Breslin PAS, Spector AC (2008). Mammalian taste perception. Curr. Biol.

[b8] Brunsø K, Fjord TA, Grunert KG (2002). http://pure.au.dk/portal/files/32302886/wp77.pdf.

[b9] Bütikofer U, Ardö Y (1999). Quantitative determination of free amino acids in cheese. Bull. Int. Dairy Fed.

[b10] Chung L, Chung SJ (2007). Cross-cultural comparisons among the sensory characteristics of fermented soybean using Korean and Japanese descriptive analysis panels. J. Food Sci.

[b11] Costell E, Tárrega A, Bayarri S (2010). Food acceptance: the role of consumer perception and attitudes. Chemosens. Percept.

[b12] Coulibaly O, Alene AD, Manyong V, Sanogo D, Abdoulaye T, Chianu J (2009). http://www.icrisat.org/what-we-do/impi/projects/tl2-publications/regional-situation-outlook-reports/rso-cwp-sbean-sub-SaharaAfrica.pdf.

[b13] CYE Consult (2009). http://www.moafsmw.org/ocean/docs/Agricultural%20Marketing/D%20Value%20chain%20Final%20Report%20Revised%2001.08.09.pdf.

[b14] Dajanta K, Apichartsrangkoon A, Chukeatirote E, Frazier RA (2011). Free-amino acid profiles of *thua nao*, a Thai fermented soybean. Food Chem.

[b15] Dajanta K, Chukeatirote E, Apichartsrangkoon A (2012). Improvement of *thua nao* production using protein-rich soybean and *Bacillus subtilis* TN51 starter culture. Ann. Microbiol.

[b16] Dakwa S, Sakyi-Dawson E, Diako C, Annan NT, Amoa-Awua WK (2005). Effect of boiling and roasting on the fermentation of soybeans into *dawadawa* (soy-*dawadawa*. Int. J. Food Microbiol.

[b17] Dooley L, Lee Y-S, Meullenet J-F (2010). The application of check-all-that-apply (CATA) consumer profiling to preference mapping of vanilla ice cream and its comparison to classical external preference mapping. Food Qual. Prefer.

[b18] Drewnowski A, Gomez-Carneros C (2000). Bitter taste, phytonutrients, and the consumer: a review. Am. J. Clin. Nutr.

[b19] Ecker O, Qaim M (2011). Analyzing nutritional impacts of policies: an empirical study for Malawi. World Dev.

[b20] Ediriweera N, Akiyama Y, Saio K (1987). Inactivation of lipoxygenase in soybeans with retention of protein solubility. J. Food Sci.

[b21] FAO (1992). Maize in human nutrition.

[b22] Golbitz P (1995). Overview of soybean processing and production. Traditional soyfoods: processing and products. J. Nutr.

[b23] Guinard J-X, Uotani B, Schlich P (2001). Internal and external mapping of preferences for commercial lager beers: comparison of hedonic ratings by consumers blind versus with knowledge of brand and price. Food Qual. Prefer.

[b24] Gupta RP (1997). Nutrition vs taste—meet the soya challenge.

[b25] Helgesen H, Solheim R, Næs T (1997). Consumer preference mapping of dry fermented lamb sausages. Food Qual. Prefer.

[b26] Jo Y-J, Cho IH, Song CK, Shin HW, Kim Y-S (2011). Comparison of fermented soybean paste (*doenjang*) prepared by different methods based on profiling of volatile compounds. J. Food Sci.

[b27] Kalimbira AA, Mtimuni BM, Mtimuni JP (2004). Effect of incorporating legumes on nutritive value of cassava-based complementary foods. Bunda J. Agric. Environ. Sci. Technol.

[b28] Katona-Apte J, Elliot V, Gillespie S (1993). Issues in food aid and nutrition. Nutritional issues in food aid—nutrition policy discussion paper no. 12.

[b29] Kim S-H, Lee K-A (2003). Evaluation of taste compounds in water-soluble extract of a *doenjang* (soybean paste). Food Chem.

[b30] Kim HG, Hong JH, Song CK, Shin HW, Kim KO (2010). Sensory characteristics and consumer acceptability of fermented soybean paste (*Doenjang*. J. Food Sci.

[b31] Kitabatake N, Gimbi DM, Oi Y (2003). Traditional non-alcoholic beverage, Togwa, in East Africa, produced from maize flour and germinated finger millet. Int. J. Food Sci. Nutr.

[b32] van Kleef E, Luning HCM, van Trijp P (2006). Internal versus external preference analysis: an exploratory study on end-user evaluation. Food Qual. Prefer.

[b33] Krokida MK, Oreopoulou V, Maroulis ZB (2000). Water loss and oil uptake as a function of frying time. J. Food Eng.

[b34] Kwon DY, Daily JW, Kim HJ, Park S (2010). Antidiabetic effects of fermented soybean products on type 2 diabetes. Nutr. Res.

[b35] Lawless HT, Heymann H (1998). Sensory evaluation of food: principles and practices.

[b36] Lawlor JB, Delahunty CM (2000). The sensory profile and consumer preference for ten speciality cheeses. Int. J. Dairy Technol.

[b37] Ley J (2008). Masking bitter taste by molecules. Chemosens. Percept.

[b38] Lindqvist M (2010). http://stud.epsilon.slu.se/2232/1/Lindqvist_M_110201.pdf.

[b39] Maleta K (2006). Undernutrition. Malawi Med. J.

[b40] Markets and Economic Research Centre of the National Agricultural Marketing Council (2011). http://www.namc.co.za/dnn/LinkClick.aspx?fileticket=hmnbvBkExdY%3D&tabid=92&mid=635.

[b41] Messina MJ (1999). Legumes and soybeans: overview of their nutritional profiles and health effects. Am. J. Clin. Nutr.

[b43] Mukai J, Tokuyama E, Ishizaka T, Okada S, Uchida T (2007). Inhibitory effect of aroma on the bitterness of branched-chain amino acid solutions. Chem. Pharm. Bull.

[b44] Murray JM, Delahunty CM (2000). Mapping consumer preference for the sensory and packaging attributes of cheddar cheese. Food Qual. Prefer.

[b45] Namgung H-J, Park H-J, Cho IH, Choi H-K, Kwon D-Y, Shim S-M (2010). Metabolite profiling of doenjang, fermented soybean paste, during fermentation. J. Sci. Food Agric.

[b46] National Statistics Office and ICF Macro (2011). Malawi demographic and health survey 2010.

[b47] National Statistics Office and Macro (2005). Malawi demographic and healthy survey 2004.

[b48] Nuss ET, Tanumihardjo SA (2010). Maize: a paramount staple crop in the context of global nutrition. Compr. Rev. Food. Sci. Food Saf.

[b49] Park S-L, Lee S-Y, Nam Y-D, Yi S-H, Lim S-I (2012). Fermentation properties of low-salted *Doenjang* supplemented with licorice and mustard. Food Sci. Biotechnol.

[b50] Parkouda C, Nielsen DS, Azokpota P, Ivetteirèneouoba L, Amoa-Awua WK, Thorsen L (2009). The microbiology of alkaline-fermentation of indigenous seeds used as food condiments in Africa and Asia. Crit. Rev. Microbiol.

[b51] Resano H, Sanjuán AI, Cilla I, Roncalés P, Albisu LM (2010). Sensory attributes that drive consumer acceptability of dry-cured ham and convergence with trained sensory data. Meat Sci.

[b52] Sarkar PK, Tamang JP (1995). Changes in the microbial profile and proximate composition during natural fermentations of soybeans to produce *kinema*. Food Microbiol.

[b53] Sarkar PK, Tamang JP, Cook PE, Owens JD (1994). *Kinema*—a traditional soybean fermented food: proximate composition and microflora. Food Microbiol.

[b54] Siegel AS, Fawcett B (1976). Food legume processing and utilization: (with special emphasis on application in developing countries).

[b55] Sripriya G, Antony U, Chandra TS (1997). Changes in carbohydrate, free amino acids, organic acids, phytate and HCl extractability of minerals during germination and fermentation of finger millet (*Eleusine coracana*. Food Chem.

[b56] Steinkraus KH (1997). Classification of fermented foods: worldwide review of household fermentation techniques. Food Control.

[b57] Thompson JL, Drake M, Lopetcharat K, Yates M (2004). Preferencce mapping of commercial chocolate milks. J. Food Sci.

[b58] Tinsley RL (2009). http://lamar.colostate.edu/~rtinsley/ValueChainAnalysisSoybeansMalawi.pdf.

[b59] Tomic O, Luciano G, Nilsen A, Hyldig G, Lorensen K, Næs T (2010). Analysing sensory panel performance in a proficiency test using the panelcheck software. Eur. Food Res. Technol.

[b60] Torres-Penaranda AV, Reitmeier CA, Wilson LA, Fehr WR, Narvel JM (1998). Sensory characteristics of soymilk and tofu made from lipoxygenase-free and normal soybeans. J. Food Sci.

[b61] United Nations Industrial Development Organization (2003). Soya bean and its recipes.

[b62] Wang H-J, Murphy PA (1996). Mass balance study of isoflavones during soybean processing. J. Agric. Food Chem.

[b63] Yamaguchi S, Ninomiya K (2000). Umami and food palatability 1. The use and utility of glutamates as flavoring agents in food. J. Nutr.

