# Sensory evaluation and consumer acceptance of naturally and lactic acid bacteria-fermented pastes of soybeans and soybean–maize blends

**DOI:** 10.1002/fsn3.120

**Published:** 2014-05-13

**Authors:** Tinna A Ng'ong'ola-Manani, Agnes M Mwangwela, Reidar B Schüller, Hilde M Østlie, Trude Wicklund

The original article to which this Corrigendum refers was published in *Food Science & Nutrition* 2(2):114–131 (DOI: 10.1002/fsn3.82).

In the published article, a funding source was omitted. The omitted information is as follows:

This research was partly financed by the International Foundation for Science (IFS) through grant no. E/4889-1 awarded to Tinna A. Ng'ong'ola-Manani.

doi: 10.1002/fsn3.120

